# Supplementary material for: SRRM2 splicing factor modulates cell fate in early development
Source: Biol Open. 2024 Apr 24;13(4):bio060415. doi: 10.1242/bio.060415 (PMC11070786; doi:10.1242/bio.060415)
Supplement: Supplementary information [file biolopen-13-060415-s1.pdf]

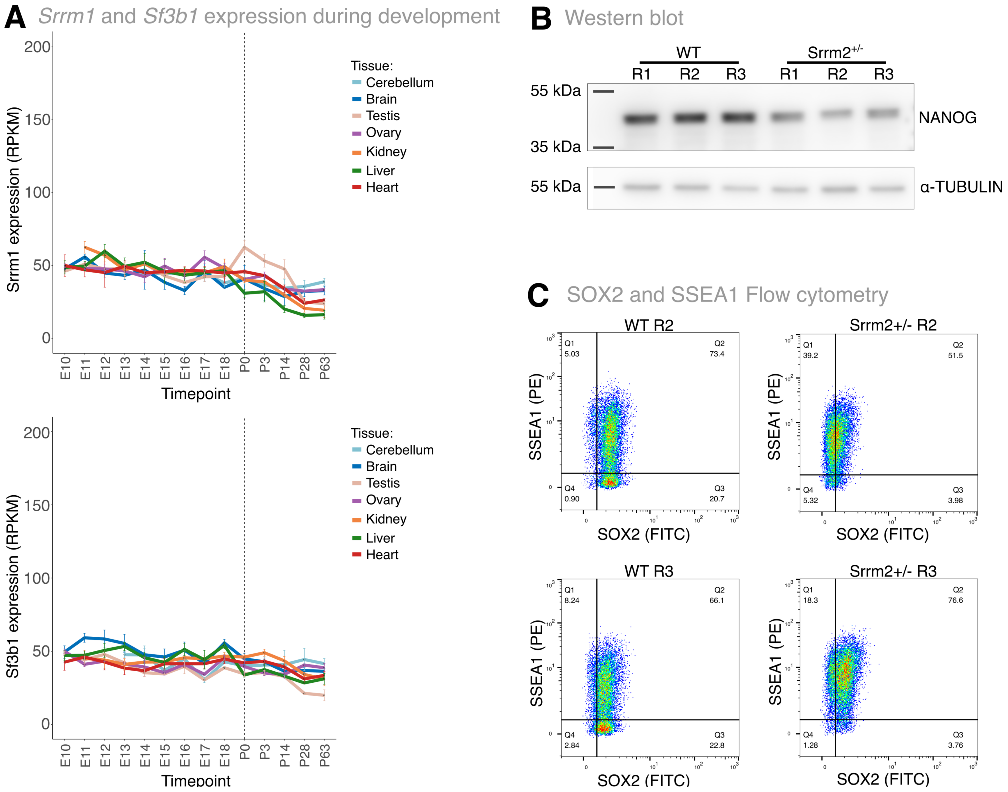

Fig. S1. *Srrm2* expression levels influence the pluripotent state of mESC. A) *Srrm2* expression levels during pre-natal (E) and post-natal (P) developing mouse tissues (RPKM) (Cardoso-Moreira et al., 2019). The vertical dashed line represents the birth time point. **B)** Western blot analysis was performed with antibodies against NANOG and α-TUBULIN, the latter was used as a loading control. Molecular weights (kDa) are shown on the left side of each blot. Three biological replicates of WT and Srrm2$^{+/-}$ cells are represented by different numbers on the top of the image. Data is representative of 2 independent experiments. **C)** Flow cytometry data of SSEA1 and SOX2 double labelling in WT and Srrm2$^{+/-}$ cells. Data is representative of 2 out of 3 biological replicates, represented by different numbers.

Biology Open • Supplementary information

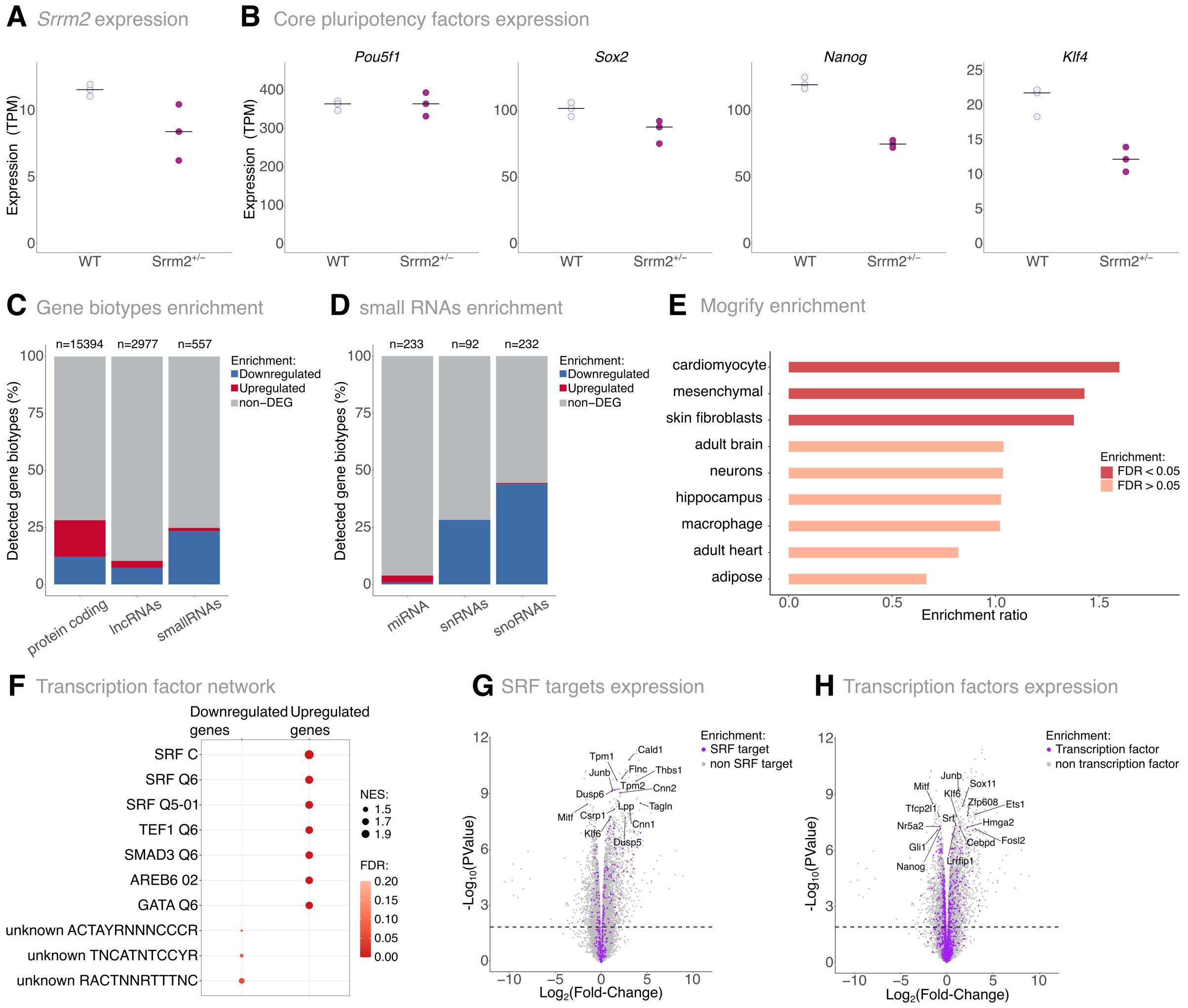

Fig. S2. ***Srrm2* heterozygous knockout mESC show extensive gene expression alterations. A)** *Srrm2* expression levels in transcript per million (TPM) per replicate in WT and Srrm2$^{+/-}$ mESC. **B)** Pluripotency transcription factors (*Pou5f1*, *Sox2, Nanog*, *Klf4*) expression levels in transcript per million (TPM) per replicate in WT and Srrm2$^{+/-}$ mESC. **C)** Proportion of downregulated (blue), upregulated (red) and non-differential expressed genes (grey) in Srrm2$^{+/-}$ cells per gene biotype. N, on top of each bar, represents the total number of detected genes per gene biotype: protein-coding (includes IG LV gene, TR V gene, protein-coding and TEC); lncRNAs (include Mt rRNA, Mt tRNA, antisense, misc RNA, polymorphic pseudogene, sense overlapping, processed transcript, processed pseudogene, pseudogene, rRNA, sense intronic, transcribed processed pseudogene, transcribed unprocessed pseudogene, translated processed pseudogene, unitary pseudogene, unprocessed pseudogene, lincRNA); and small RNAs (include miRNA, snRNA and snoRNA). **D)** Proportion of downregulated (blue), upregulated (red) and non-differential expressed small RNAs (grey) in Srrm2$^{+/-}$ cells. N, on top of each bar, represents the total number of detected genes per small RNAs biotype. **E)** Over-representation analysis (ORA) for the publicly available data of transdifferentiation transcription factors imputed by Mogrify algorithm (Rackham et al., 2016; Ferrai et al., 2017) using the identified upregulated genes in Srrm2$^{+/-}$ mESC. Different red colours represent FDR values for each enrichment term. **F)** GSEA for mouse transcription factors of Srrm2$^{+/-}$ vs WT gene expression comparison. Red colours represent the FDR values, whereas the circle size indicates the Normalized Enrichment Score (NES) for each set of transcription factor gene targets. **G)** Volcano plot showing the expression of the SRF transcription factors targets (purple) in the transcriptional comparison of Srrm2$^{+/-}$ and WT mESC. Gene labels represent the top 15 SRF gene targets with the lowest adjusted P-value. **H)** Volcano plot showing the expression of all *Mus musculus* transcription factors (purple) in the transcriptional comparison of Srrm2$^{+/-}$ and WT mESC. Gene labels represent the top 15 transcription factor encoding genes with the lowest adjusted P-value.

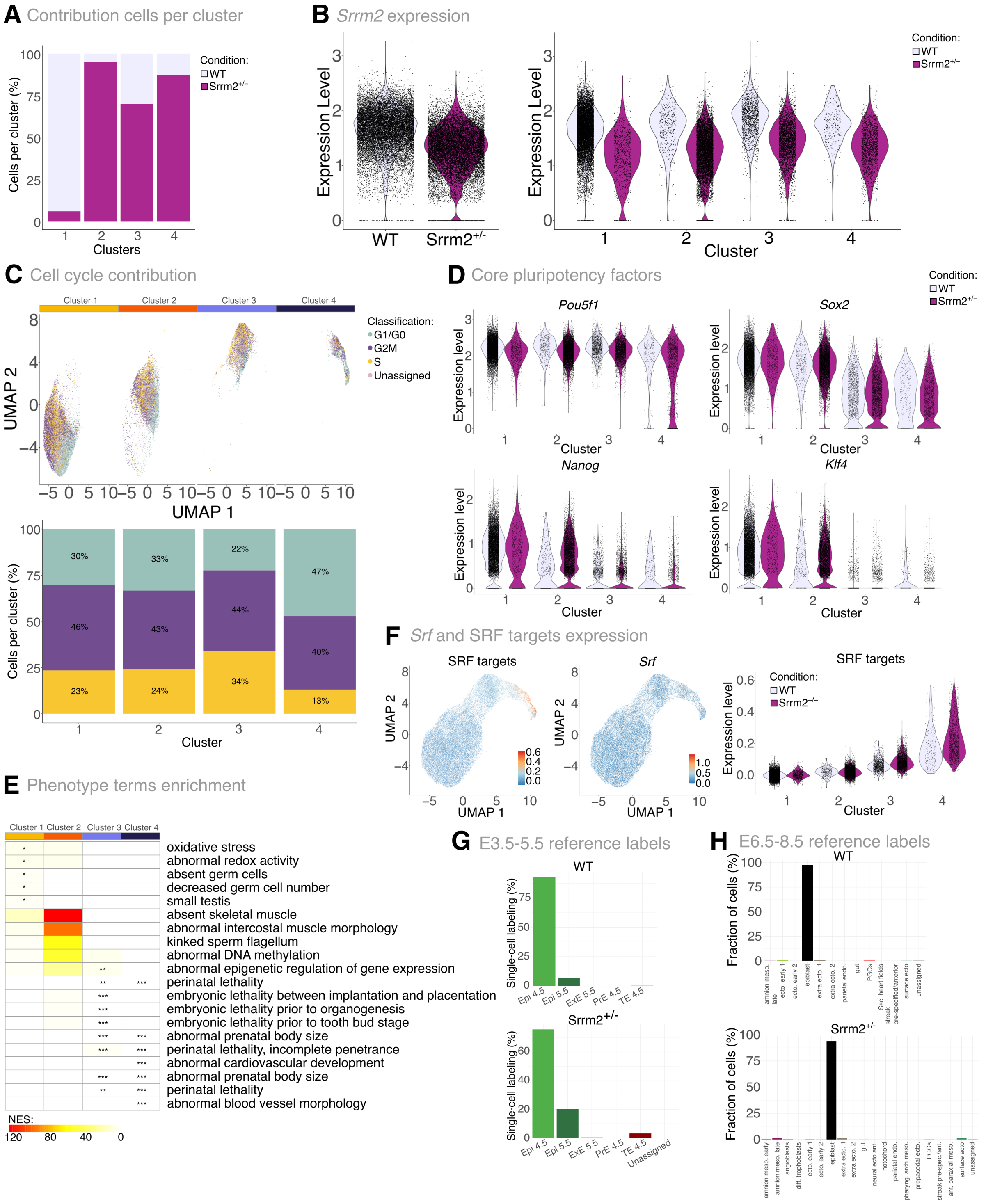

Fig. S3. Single-cell transcriptome alterations in Srrm2 heterozygous knockout ESCs. A) Proportional contribution of WT (light pink) and Srrm2$^{+/-}$ (dark pink) cells per cluster. **B)** Single-cell distributions of *Srrm2* expression levels per condition (left) and cluster (right). Each dot represents a single cell. Conditions are represented in different colours: WT (light pink) and Srrm2$^{+/-}$ ESC (dark pink). **C)** Contribution of cell cycle phases. On top: UMAP projection of cell cycle phases per cell. Cells are split by cluster, identified by different colours on top. Cells are coloured by cell cycle phases. Below: Proportional contribution of cell cycle phases per cluster. **D)** Single-cell distributions of the expression levels of pluripotency transcription factors (*Pou5f1*, *Sox2*, *Nanog*, *Klf4*) per condition and cluster. Each dot represents a single cell. Conditions are represented in different colours: WT (light pink) and Srrm2$^{+/-}$ ESC (pink). Clusters are distinguished in the y-axis. **E)** Heatmap representing phenotype terms enrichment from ORAs analyses of clusters gene markers

Biology Open • Supplementary information

per cluster. The top 5 significant terms per cluster are displayed. Clusters are represented by different colours on the top (from left to right, clusters 1 to 4). Normalised enrichment scores (NES) of GO terms are represented by different colours (yellow to red). Significant NES values are marked with an asterisk (Fisher exact test *: adjusted P-value < 0.05; **: adjusted P-value < 0.01; ***: adjusted P-value < 0.001). **F)** Expression of SRF gene targets or *Srf*. On the left: UMAPs representing the relative similarity between individual cells coloured by the average expression levels of SRF gene targets (left) or by *Srf* (right). Right: Single-cell distributions of SRF-target genes average single-cell expression per condition and cluster. Each dot represents a single cell. Conditions are represented in different colours: WT (light pink) and Srrm2$^{+/-}$ ESC (pink). Clusters are distinguished in the y-axis. **G-H)** Proportion of WT (top) and Srrm2$^{+/-}$ (down) cells assigned to predicted labels from *in vivo* scRNA-seq reference atlas of mouse embryogenesis cells from E3.5- 5.5 (Nowotschin et al., 2019) (G) and E6.5-8.5 (Chan et al., 2019; Grosswendt et al., 2020, Haggerty et al., 2021) (H). G) Cells are classified into epiblast 4.5 (Epi 4.5), epiblast 5.5 (Epi 5.5), extra-embryonic ectoderm (ExE 5.5), primitive endoderm 4.5 (PrE 4.5), trophoectoderm 4.5 (TE 4.5). H) Cells are classified into amnion mesoderm early, amnion mesoderm late, angioblasts, differentiated trophoblasts, ectoderm early 1, ectoderm early 2, epiblast, extraembryonic ectoderm 1 (ExE 1), extraembryonic ectoderm 2 (ExE 2), gut, neural ectoderm anterior, notochord, parietal endoderm, pharyngeal arch mesoderm, primordial germ cells (PGCs), preplacodal ectoderm, surface ectoderm, secondary heart field/splanchnic lateral plate, splanchnic-lateral/anterior-paraxial mesoderm, streak pre-specified/anterior, unassigned (H).

Biology Open • Supplementary information

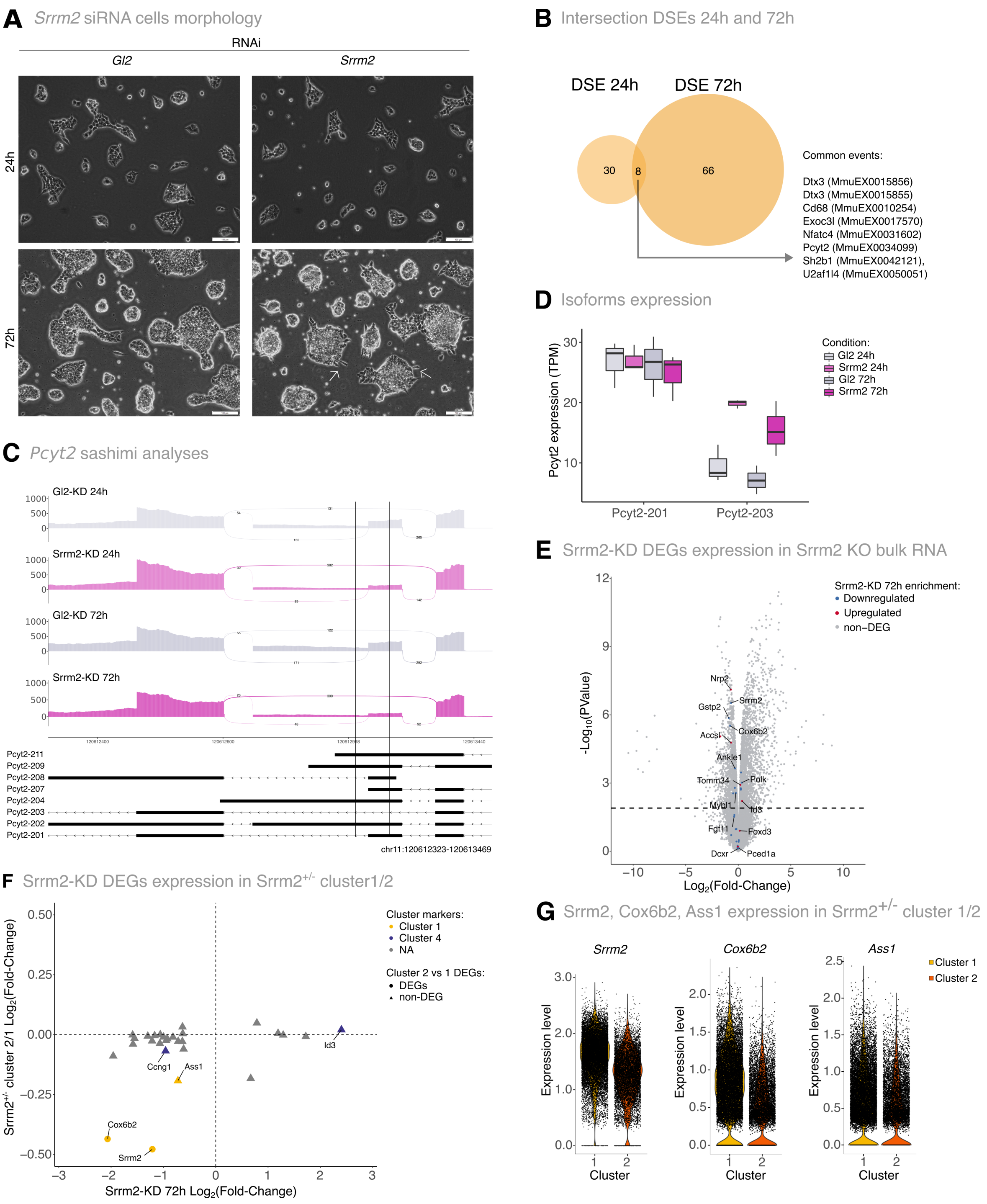

Fig. S4. Transcriptome alterations upon *Srrm2* RNAi knockdown. A) Brightfield images from mESC cultures treated with Gl2 (control) or with *Srrm2* RNAi, at 24h or 72h. Images are representative of 4 independent experiments. Scale bar corresponds to 100 µm. Arrows indicate changes in colony morphology that are noticeable at 72h post siRNA treatment. **B)** Venn diagram of the differentially spliced events at 24h and 72h. The genes and the splice events of the eight common differentially spliced events between 24h and 72h are listed. **C)** Sashimi plots displaying the differential splicing events for the *Pcyt2* gene region (chr11:120612323-120613469) in control samples and *Srrm2* knockdown samples at 24h and 72h. Splicing events are delimited with vertical black lines (*Pcyt2*: chr11:120613030-120613083). Numbers in the plot represent the average number of junction reads from three replicates. Isoforms that cover the represented genomic region are shown and identified as following: ENSMUST00000145356 (Pcyt2-211), ENSMUST00000142049

(Pcyt2-209), ENSMUST00000134671 (Pcyt2-208), ENSMUST00000126723 (Pcyt2-207), ENSMUST00000124468 (Pcyt2-204), ENSMUST00000106188 (Pcyt2-203), ENSMUST00000106187 (Pcyt2-202), ENSMUST00000026129 (Pcyt2-201). **D)** Expression levels (TPM) of *Pcyt2* isoforms upon Gl2 (control) and *Srrm2* knockdown at 24h and 72h. Isoforms with expression levels above 5 TPM and encoding for proteins are displayed. **E)** Volcano plot of Srrm2$^{+/-}$ and WT transcriptional comparison with differentially expressed genes in the *Srrm2* knockdown being represented in different colours. Gene labels represent the top-10 *Srrm2* knockdown differentially expressed genes with the lowest adjusted P-value at 72h and genes mentioned in the text (*Id3*, *Nrp2*, *Fgf11*). **F)** Expression comparison (Log_2 Fold change) of the differentially expressed genes upon 72h of *Srrm2* knockdown with the most stem-like single-cell clusters. Negative values represent downregulated genes in *Srrm2*-depleted cells and positive values represent upregulated genes in *Srrm2*-depleted cells. Gene markers of single-cell clusters are represented by different colours. Differentially expressed genes of the most stem-like single-cell clusters (cluster 2 vs cluster 1) are represented by different shapes. **G)** Single-cell distributions of the expression levels of *Srrm2*, *Cox6b2* and *Ass1* per condition and cluster. Each dot represents a single cell. Conditions are represented in different colours: WT (light pink) and Srrm2$^{+/-}$ cells (pink). Clusters are distinguished in the y-axis.

Biology Open • Supplementary information

Table S1. Differentially Expressed Genes between Srrm2+/- vs WT ES cells

Available for download at
https://journals.biologists.com/bio/article-lookup/doi/10.1242/bio.060415#supplementary-data

Table S2. Differentially Expressed Genes between Srrm2+/- vs WT ES cells

Available for download at
https://journals.biologists.com/bio/article-lookup/doi/10.1242/bio.060415#supplementary-data

Table S3. GSEA single-cell embryo cell lineage markers in Srrm2+/- cells

Available for download at
https://journals.biologists.com/bio/article-lookup/doi/10.1242/bio.060415#supplementary-data

Table S4. ORA mogrify transcription factors in Srrm2+/- cells

Available for download at
https://journals.biologists.com/bio/article-lookup/doi/10.1242/bio.060415#supplementary-data

Table S5. GSEA transcription factor network in Srrm2+/- cells

Available for download at
https://journals.biologists.com/bio/article-lookup/doi/10.1242/bio.060415#supplementary-data

Table S6. Signigicant differential splicing events in Srrm2+/- cells

Available for download at
https://journals.biologists.com/bio/article-lookup/doi/10.1242/bio.060415#supplementary-data

Table S7. Differential expressed genes for cluster 2 vs cluster 1

Available for download at
https://journals.biologists.com/bio/article-lookup/doi/10.1242/bio.060415#supplementary-data

Table S8. Single-cell gene markers per cluster

Available for download at
https://journals.biologists.com/bio/article-lookup/doi/10.1242/bio.060415#supplementary-data

Table S9. ORA biological terms for single-cell clusters markers

Available for download at
https://journals.biologists.com/bio/article-lookup/doi/10.1242/bio.060415#supplementary-data

Table S10. ORA phenotype terms for single-cell cluster markers

Available for download at
https://journals.biologists.com/bio/article-lookup/doi/10.1242/bio.060415#supplementary-data

Table S11. Significant differential splicing events in Srrm2KD 24H cells

Available for download at
https://journals.biologists.com/bio/article-lookup/doi/10.1242/bio.060415#supplementary-data

Table S12. Significant differential splicing events in Srrm2KD 72H cells

Available for download at
https://journals.biologists.com/bio/article-lookup/doi/10.1242/bio.060415#supplementary-data

Table S13. Expression of transcript isoforms in Srrm2KD 24H and 72H replicates

Available for download at
https://journals.biologists.com/bio/article-lookup/doi/10.1242/bio.060415#supplementary-data

Table S14. Gene expression in Srrm2KD 24H cells

Available for download at
https://journals.biologists.com/bio/article-lookup/doi/10.1242/bio.060415#supplementary-data

Table S15. Gene expression in Srrm2KD 72H cells

Available for download at
https://journals.biologists.com/bio/article-lookup/doi/10.1242/bio.060415#supplementary-data

Table S16. Antibodies used in this study

Available for download at
https://journals.biologists.com/bio/article-lookup/doi/10.1242/bio.060415#supplementary-data
